# Profiles and Predictive Values of Interleukin-6 in Aortic Dissection: a Review

**DOI:** 10.21470/1678-9741-2018-0287

**Published:** 2019

**Authors:** Shi-Min Yuan

**Affiliations:** 1 Department of Cardiothoracic Surgery, The First Hospital of Putian, Teaching Hospital, Fujian Medical University, Putian, Fujian Province, People's Republic of China.

**Keywords:** Aneurysm, Dissecting, Inflammation, Interleukin-6

## Abstract

Aortic dissection (AD) has been recognized to be associated with an inflammatory process. Clinical observations demonstrated that patients with AD had an elevated interleukin (IL)-6 level in comparison to hypertensive or healthy controls. Adverse events such as acute lung injury, postimplantation syndrome, and death are associated with an elevated IL-6 level. Thus, circulating IL-6 could be a reliable biomarker for the diagnosis of AD and for the eveluation of the therapeutic outcomes and the prognosis of AD patients. Therapeutic interventions aiming at attenuating the inflammatory status by IL-6 neutralization could effectively decrease the IL-6 level and thus reverse the progression of the disorder of AD patient. Endovascular aortic repair can effectively control the inflammatory cytokines. Selective antegrade cerebral perfusion with deep hypothermic circulatory arrest during aortic arch replacement shows better neuroprotectve effect with an improved IL-6 level of the cerebrospinal fluid. These results facilitate the understanding of the etiology of AD and guide the directions for the treatment of acute AD in the future. More effective therapeutic agents developed based on the theories of IL-6 signaling involved in the mechasims of AD are anticipated.

**Table:** 

Abbreviations, acronyms & symbols	
AAD	= Acute aortic dissection
AD	= Aortic dissection
ALI	= Acute lung injury
CAD	= Chronic aortic dissection
DHCA	= Deep hypothermic circulatory arrest
ELISA	= Enzyme linked immunosorbent assay
HTN	= Hypertension
IL	= Interleukin
MCP-1	= Monocyte chemoattractant protein-1
PIS	= Postimplantation syndrome
POD	= Postoperative day
SACP	= Selective antegrade cerebral perfusion
TNF-α	= Tumor necrosis factor alpha

## INTRODUCTION

The interleukin (IL) family is a group of cytokines involved in the pathogenesis of inflammatory, allergic, infectious, immunodeficient, neoplastic, fibrotic, and hypoxic disorders^[1,2]^. Of the cardiac surgical patients, those with aortic dissection (AD) showed particular inherent relationships to IL-6^[3]^. Clinical research revealed, in patients receiving an aortic aneurysm/dissection repair, that the circulating IL-6 levels differed between acute AD and hypertensive or healthy controls^[3]^. However, there has been no report comprehensively describing the profiles of circulating IL-6 levels in patients with AD so far. In order to highlight the potential role of the circulating IL-6 in patients with AD, a comprehensive review is conducted.

## METHODS

PubMed and "*Baidu*" Scholar databases were carefully retrieved for publications reporting on IL-6 in AD patients, published between 2000 and 2017. The search terms included "interleukin-6", "aortic dissection", and "circulating/serum/plasma/blood/cerebrospinal fluid". Bibliographic references were also tracked down for the completeness of the literature retrieval. Immunohistochemistry of IL-6 in AD patients were not included in this study.

Data were carefully extracted for details of the study population, demographics of patients, type of AD, detection method and unit of IL-6, source of sampling, circulating IL-6 values, and therapeutic interventions.

Quantitative data were presented as mean±standard deviation with range and median values. The intergroup differences were compared by independent samples *t*-test. *P*<0.05 was considered statistically significant.

## RESULTS

### Patient Information

In total, 19 articles were collected^[4-22]^. The study group included 787 AD patients. In one (5.3%) of the reports, the study group involved both acute and subacute AD cases, but the authors did not indicate the patient number of different AD phases^[21]^. Therefore, the prevalence of acute and subacute AD cases in the study groups could not be assessed. The control groups comprised 1,164 patients, including 543 AD patients (461 acute AD, 47 subacute AD, and 35 chronic AD patients), 260 hypertensive patients, and 361 healthy individuals (Table 1).

**Table 1:** Patients’ settings of the two groups.

Patients’ setting	Study Group	Control
Aortic dissection	787 (100)	543 (46.6)
Type A	315 (40.0)	191 (35.2)
Type B	303 (38.5)	305 (56.2)
Type unspecified	169 (21.5)	47 (8.7)
Hypertension		260 (22.3)
Healthy		361 (31.0)

There was no significant difference in the patients’ ages between the study and control groups (53.3±6.1 years *vs. *51.2±5.7 years, *P*=0.2702). In the study groups, there were 685 (72.6%) male and 259 (27.4%) female patients with a male-to-female ratio of 2.64:1, while in the control groups, there were 700 (69.9%) male and 301 (30.1%) female patients with a male-to-female ratio of 2.33:1.

Apart from the observations on the surgical or endovascular treatment and adverse events, such as acute lung injury (ALI)^[8]^, postimplantation syndrome (PIS)^[5]^, and death^[16]^, therapeutic interventions in the study groups also included dexmedetomidine^[4]^, 50% xenon^[23]^, antithrombin^[10]^, deep hypothermic circulatory arrest (DHCA) with selective antegrade cerebral perfusion (SACP)^[13]^, ulinastatin^[14]^, and *Qishen Yiqi *dripping pill, a Chinese patent drug^[12]^.

Most of the samples for IL-6 detection were drawn from the veins, especially from the peripheral veins, while samples of a few patients were drawn from the artery or cerebrospinal fluid (Table 2). In six (31.6%) reports, the detection of IL-6 was not mentioned. Of the remaining 13 (68.4%) reports, IL-6 was detected by enzyme linked immunosorbent assay (ELISA) in 12 (92.3%) reports^[4,6,9,10,14-16,18-21]^, and in one (7.7%) report, the detection method was not given, but an Immulite 2000 System Analyzer for this purpose was described, instead^[5]^. In 17 reports, the unit of IL-6 was pg/mL or ng/L^[4-7,9-13,15-21]^, whereas in two reports, it was ug/L^[8,14]^. The detection method of IL-6 in the latter two reports was not described.

**Table 2:** Source of samples.

Source of sample	Study Group	Control	Total
Vein	563 (95.1)	684 (95.5)	1247 (95.8)
Peripheral	294 (52.2)	363 (53.1)	657 (52.7)
Central	113 (20.1)	119 (17.4)	232 (18.6)
Unspecified	156 (27.7)	202 (29.5)	358 (28.7)
Artery	20 (3.3)	20 (2.8)	40 (3.1)
Cerebrospinal fluid	9 (1.5)	5 (0.7)	14 (1.1)

### The Effect of Pathology before Treatment

**Fig. 1:**
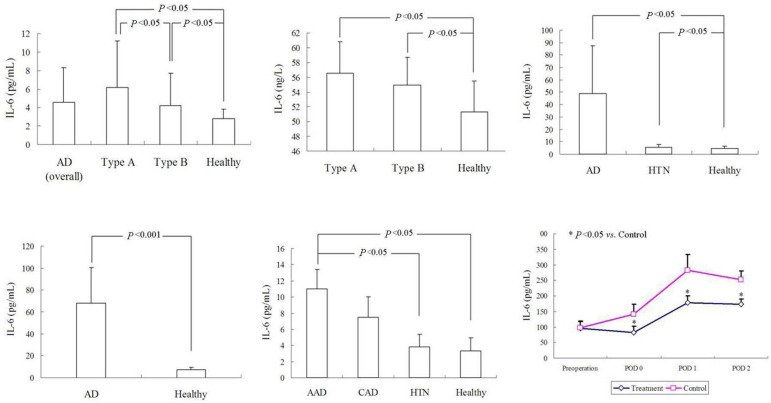
The interleukin-6 (IL-6) levels of patients with aortic dissection (AD) in comparison to those of the controls: (A) IL-6 levels of patients with acute type A AD were significantly higher than those of healthy controls; (B) IL-6 levels of either type A or type B AD patients were higher than those of healthy controls; (C) IL-6 levels between AD patients and hypertensive or healthy subjects; (D) patients with type A AD showed a much higher IL-6 level than controls; (E) IL-6 values were higher in patients with acute aortic dissection (AAD) than in hypertensive or healthy subjects; and (F) deceased patients with AAD showed a higher plasma IL-6 level than survival patients. CAD = chronic aortic dissection; HTN = hypertension; POD = postoperative day.

Circulating IL-6 levels of acute type A AD were significantly higher than those of the healthy control (Figure 1A), and so were other proinflammatory cytokines, such as IL-4 and tumor necrosis factor alpha (TNF-α)^[19]^. Li et al.^[12]^ reported that in patients with AD, of either type A or type B, the IL-6 level was higher than in the healthy control (Figure 1B). Zhong et al.^[21]^ compared IL-6 levels between AD patients and hypertensive or healthy subjects. They noted that AD patients had a much higher IL-6 level than the other two groups (Figure 1C), whereas the sampling time was not mentioned. Nevertheless, the treatment of choice was not mentioned. Yang and Meng^[20]^ found out that type A AD patients had a much higher IL-6 level than the control patients (Figure 1D). However, the sampling time broadly extended from one day to 14 days after the onset of AD, and the treatment of choice was not stated at all in their report. The plasma TNF-α and IL-6 levels were measured by Qin et al.^[15]^ and they showed that the IL-6 level was significantly higher in the AD group than in the other two groups (175±38 *vs*. 50±8 *vs*. 50±7 pg/mL, *P*<0.05). A similar trend was also reported by Gu et al.^[6]^, who described that the IL-6 level was significantly higher in patients with type A AD than in those with uncontrolled hypertension (50.41±42.95 pg/mL *vs*. 5.82±2.49 pg/mL, *P*<0.05) and control groups (50.41±42.95 pg/mL *vs*. 4.42±2.12 pg/mL, *P*<0.05). A similar significant increase of the plasma TNF-α level was found in type A AD patients. The time intervals to the peak plasma levels of IL-6 and TNF-α were shorter than of the C-reactive protein^[6]^, indicating that IL-6 and TNF-α were more sensitive than C-reactive protein. Wen et al.^[18]^ reported that the IL-6 value was higher in patients with acute AD than in hypertensive patients and healthy controls (10.98±2.38 *vs*. 3.79±1.56 *vs*. 3.32±1.60 pg/mL, *P*<0.05) (Figure 1E). No relationships were found between IL-6, C-reactive protein, TNF-α, matrix metalloproteinase-9 concentrations, and type of AD. In their "Methods" section, the authors described three sampling time points; however, in their "Results" section, only one set of IL-6 values was reported, and, for this set, the sampling time was not indicated. It was presumably the preoperative value^[18]^. The deceased group of acute AD patients showed a higher plasma IL-6 level than the survival patients (17.92±4.61 pg/mL *vs*. 12.59±2.53 pg/mL, *P*<0.001) (Figure 1F), indicating that IL-6 could be a predictive biomarker for mortality, and the cutoff value for the prediction of death should be 18.36 pg/mL, with a sensitivity of 87.4% and a specificity of 70.8%^[16]^.

**Fig. 2:**
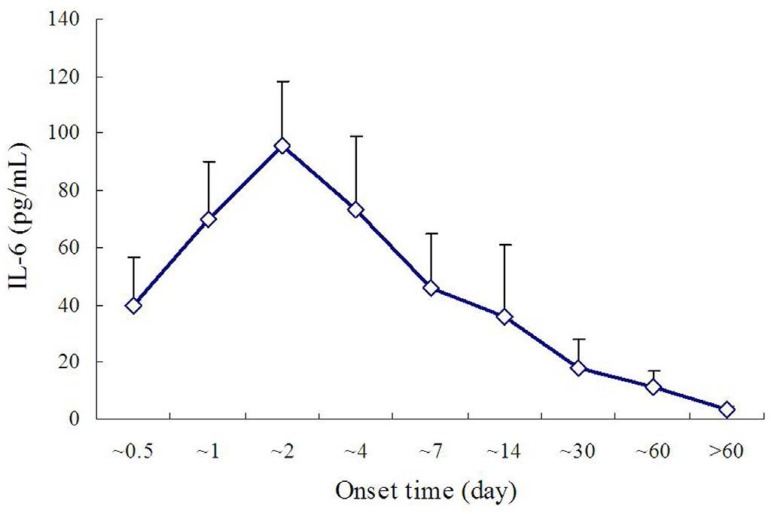
Dynamic changes of interleukin-6 (IL-6) value showed a gradual increase and it reached a peak value on day 1-2 after the onset of aortic dissection

Zhong et al.^[21]^ also reported the dynamic changes of IL-6 in AD group patients. They observed that, with the development of AD, IL-6 values increased gradually and reached a peak value on days 1-2 after the onset, followed by a gradual decrease. The IL-6 value recovered to normal range in two months (Figure 2).

### The Effect of Conservative Treatment

**Fig. 3:**
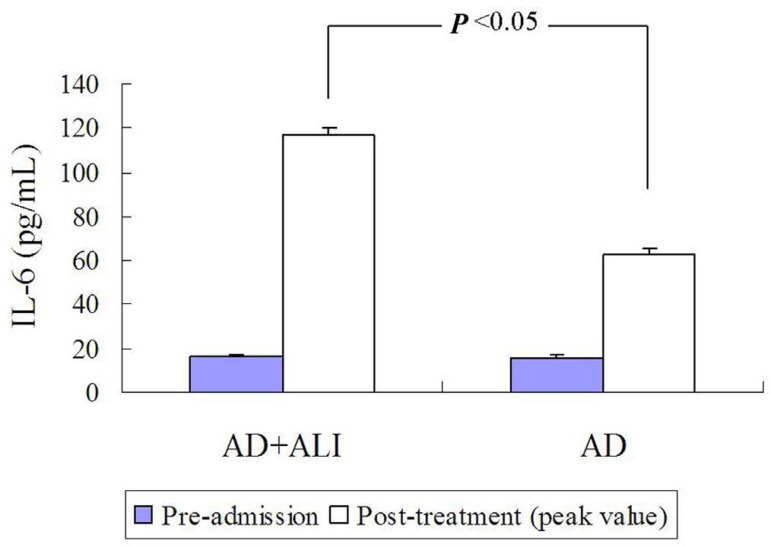
The interleukin-6 (IL-6) levels were significantly higher in the acute lung injury (ALI) group than in the non-ALI group. AD = aortic dissection.

According to the oxygen index (PaO_2_/FiO_2_) under static oxygen inhalation, Huang et al.^[8]^ divided their patients with AD into two groups: the ALI group (PaO_2_/FiO_2_ ≤200 mmHg) (*n*=26) and the non-ALI group (PaO_2_/FiO_2_ >200 mmHg) (*n*=59). On admission, no difference was noted in IL-6 levels between the two groups. During the conservative treatment, IL-6 and IL-8 initially increased and peaked in 48 hours, and then decreased gradually. IL-6 levels were significantly higher in the ALI group than in the non-ALI group (116.4±4.1 µg/L *vs*. 62.9±2.3 µg/L, *P*<0.05) (Figure 3)^[8]^.

### The Effect of Endovascular Aortic Repair

**Fig. 4:**
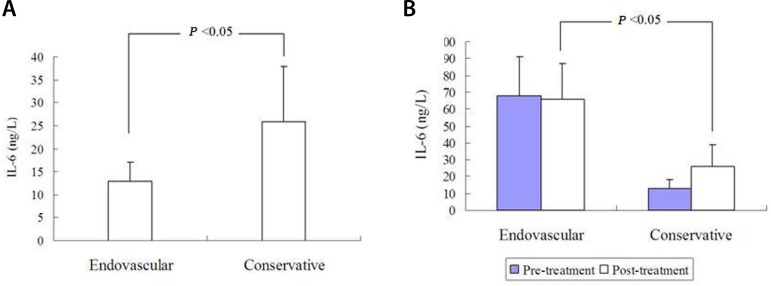
The interleukin-6 (IL-6) level of aortic dissection patients with acute lung injury: (A) IL-6 level of endovascularly treated patients was lower than of the medically treated patients; and (B) IL-6 level of endovascularly treated patients after treatment (time after treatment was not described) was lower than of the medically treated patients. No difference of IL-6 level was found between the groups before treatment.

**Fig. 5:**
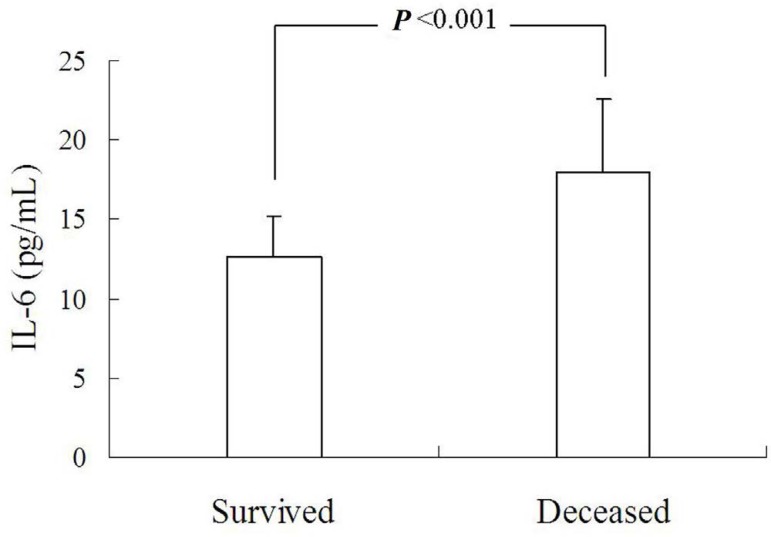
The interleukin-6 (IL-6) level was significantly higher in the patients with postimplantation syndrome (PIS) group than in those without it.

**Fig. 6:**
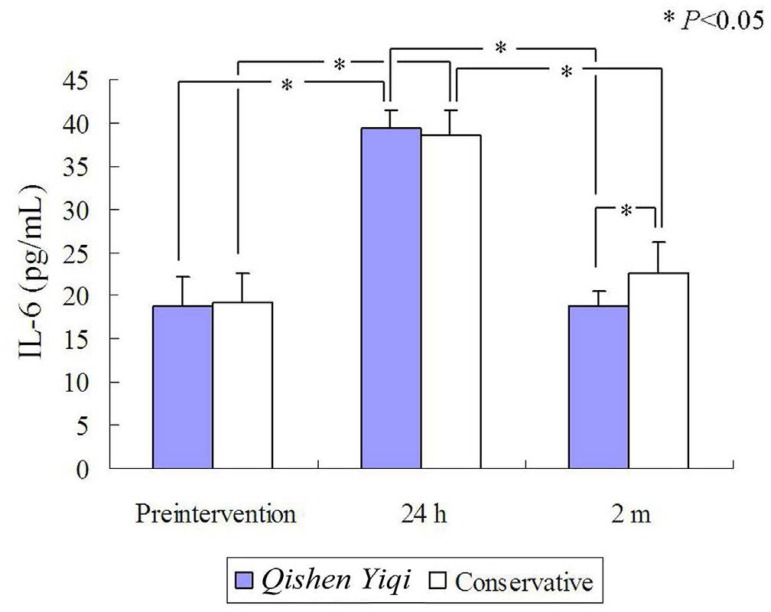
Patients who received Qishen Yiqi pills had a significant lower serum interleukin-6 (IL-6) level than those who received conventional therapy.

Guo et al.^[7]^ divided their 187 acute AD patients with ALI randomly into two groups according to treatment of choice: 94 were treated endovascularly with a stent graft and 93 were treated medically. In the endovascularly treated patients, the IL-6 level was lower than in the medically treated patients (13±4 ng/L *vs*. 26±12 ng/L, *P*<0.05) (Figure 4A). Li et al.^[10]^ performed a similar study by dividing 60 patients with acute type B AD with ALI randomly into two groups: the observation group (30 patients receiving conservative and endovascular treatments) and the control group (30 patients receiving conservative treatment only). It showed that IL-6 levels did not differ before treatment, and the observation group showed a significantly lower IL-6 level after treatment (time after treatment was not described) than the control group (13.4±5 ng/L *vs*. 26.4±13 ng/L, *P*<0.05) (Figure 4B). The total effective rate of the observation group was 100% (30/30), higher than the 73.3% (22/30) of the control group^[11]^. Gorla et al.^[5]^ reported that PIS was diagnosed in 15.8% of AD patients receiving endovascular aortic repair. The IL-6 levels significantly increased in the PIS group, and peaked 24 hours after endovascular aortic repair (Figure 5)^[5]^. For Debakey type III AD patients after endovascular aortic repair, routine treatment (β-blockers, sodium nitroprusside, non-dihydropyridine calcium channel blocker, and angiotensin converting enzyme inhibitor) combined with *Qishen Yiqi* pills can lower the serum levels of inflammatory cytokines, including IL-6 (Figure 6) and TNF-α^[21]^.

### The Effect of CPB and Heart Arrest or Selective Cerebral Perfusion

**Fig. 7:**
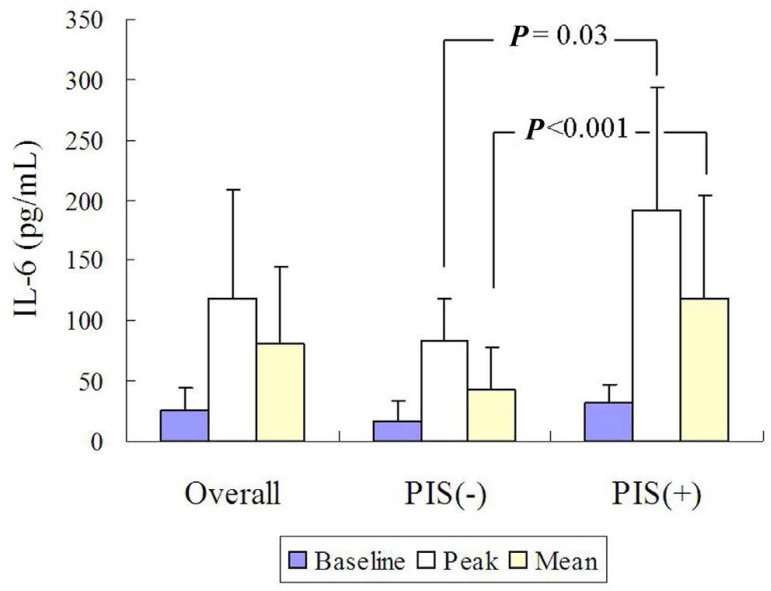
Deep hypothermic circulatory arrest (DHCA) patients showed higher levels of interleukin-6 (IL-6) with double peaks during the perioperative period, whereas the combined DHCA and selective antegrade cerebral perfusion (SACP) patients showed lower IL-6 levels with a single early peak at the end of the operation. AD=aortic dissection.

Differences were noted in IL-6 levels of the cerebrospinal fluid between surgically treated AD patients under either sole DHCA or combined DHCA and SACP; patients with DHCA showed higher IL-6 levels with double peaks during the perioperative period, whereas patients with combined DHCA and SACP showed lower IL-6 levels with a single early peak at the end of the operation (Figure 7)^[13]^.

### The Effect of Drugs/Agents

**Fig. 8:**
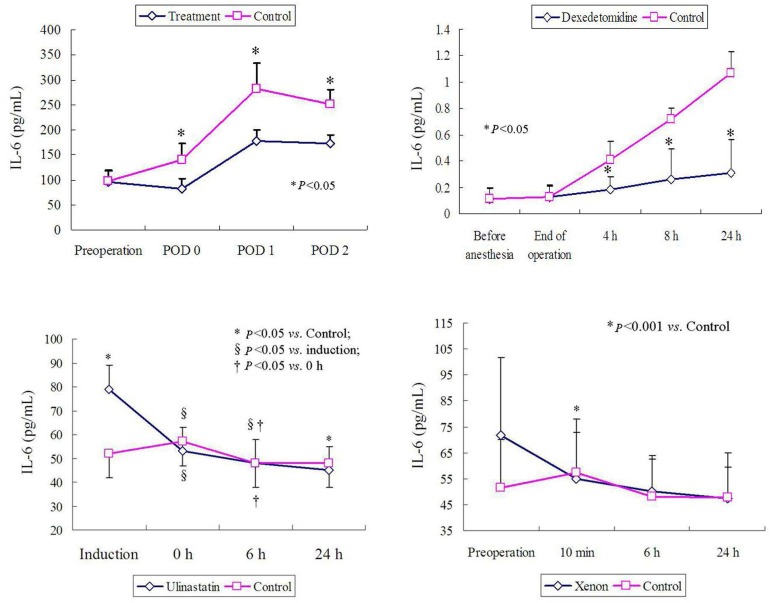
Impacts of drug therapies on interleukin-6 (IL-6) levels of aortic dissection patients: (A) the infusion of antithrombin (3000 U) significantly inhibited the inflammatory situation, leading to a significantly decreased IL-6 level; (B) IL-6 values were significantly lower than those without dexmedetomidine use 4, 8, and 24 hours after the operation; (C) a comparison of dynamic IL-6 levels of acute aortic dissection patients undergoing surgical treatment between those with or without the use of ulinastatin. The ulinastatin patients showed a stepwise reduction of IL-6 levels from the start of operation to 24 hours after operation; and (D) patients treated with xenon had higher levels of IL-6 compared to the control group before surgery. h=hour; POD=postoperative day

A clinical observation demonstrated that the infusion of antithrombin (3000 U) significantly inhibited the inflammatory situation, leading to a significantly decrease in IL-6 level (Figure 8A)^[10]^. In acute AD patients receiving total arch replacement administered with dexmedetomidine during the perioperative period, the IL-6 values were significantly lower than in those without dexmedetomidine use 4, 8, and 24 hours after the operation (Figure 8B)^[4]^. A comparison of dynamic IL-6 levels of acute AD patients undergoing surgical treatment between those with or without the use of ulinastatin revealed that the ulinastatin group showed a stepwise reduction of IL-6 levels from the start of operation to 24 hours after operation (Figure 8C)^[14]^.

Pulmonary static inflation with 50% xenon during cardiopulmonary bypass attenuated the decreased oxygen index and increased the respiratory index values at the end of operation for Stanford type A AD. Patients treated with xenon had higher levels of IL-6 compared to the control group before surgery (Figure 8D). In the second (postoperative 10 minutes to postoperative six hours) and third fractions (postoperative 6-24 hours), IL-6, IL-10, TNF-α, and thromboxane B_2_ levels were similar in both groups^[9]^.

Sato et al.^[17]^ reported that a 67-year-old female patient was diagnosed with advanced stage lung adenocarcinoma and she was started on chemotherapy with 3.6 mg of pegfilgrastim as primary prophylaxis for neutropenia. The pegfilgrastim use led to the development of thoracic aortitis and subsequent asymptomatic AD. The authors stated that the elevated serum IL-6 level observed in this patient might be a cause of the occurrence of aortic disorders. However, the authors did not mention the subsequent management of the associated AD^[17]^.

As a result of blood test in AD patients, the elevations of IL-6, IL-8, and granulocyte-colony stimulating factor were observed after the onset of AD, therefore the mechanism of vascular inflammation after AD was common in a mouse model of AD and in patients with acute AD, suggesting that the IL-8 receptor antagonist (under development) and the anti-human IL-6 monoclonal antibody preparation may be effective in preventing the complications and improving the prognosis of patients with acute AD^[24]^.

## DISCUSSION

The significantly increased levels of the proinflammatory cytokines in acute AD patients illustrated that the occurrence of AD was closely related to the inflammatory condition and stress of the patient^[19]^. IL-6 is a pyrogen and is in a close relation to the constitutional symptom, such as fever, of AD patients. Plasma IL-6 reaches a peak value within 1-2 hours after the onset of AD. It is hypothesized that there is usually an uncontrolled hypertension at the initial stage of AD onset, and that IL-6 is also involved in the regulation of blood pressure^[21]^. However, it is unclear that the significant difference found in circulating IL-6 between hypertensive patients and healthy individuals should be explained by the fact that the blood pressure of hypertensive patients was not well controlled.

Guo et al.^[7]^ tried to explain the associated ALI in AD patients. They proposed that the activation of inflammatory cells and the release of inflammatory mediators might occur in acute AD patients. The pulmonary function of the patients was therefore compromised, and it thus led to ALI. Endovascular repair of AD could effectively control the inflammatory cytokines and the aorta was well remodeled.

IL-6 is released by the vascular endothelium in the blood stream and stimulates the liver to produce acute phase proteins, such as fibrinogen and C-reactive protein. In a study involving 22 patients receiving endovascular aortic repair, the inflammatory cascade is initiated by IL-6 release from aneurysmal thrombus formation, resulting in the synthesis of TNF-α^[5]^. IL-6 may play a pivotal role in the pathogenesis of PIS, which represents a systemic inflammatory response syndrome initially observed following endovascular aortic repair of infrarenal abdominal aortic aneurysms. All-cause mortality occurred in 6.3% (7/112) of the non-PIS patients and in 0.0% (0/21) of the PIS patients. The predictive value of IL-6 in the postoperative mortality was not evaluated^[5]^.

Cardiopulmonary bypass-induced leucocytosis and increased plasma IL-6 and TNF-α values indicate an associated inflammatory response. Experimental studies revealed that the systemic inflammatory response syndrome did not seem to be provoked during DHCA and was mainly induced during reperfusion^[25]^. Clinical observations also disclosed that the IL-6 level was much lower in DHCA patients than in patients with low-flow cardiopulmonary bypass 0.5 and 2 hours after operation^[26]^. SACP appeared to be superior to hypothermic circulatory arrest alone in terms of neuroprotective effects^[23]^. It allowed more complicated arch repair procedures to be performed with a significantly longer cerebral exclusion time without increasing the risks of stroke or death^[27]^.

Ulinastatin can inhibit the release of the inflammatory cytokines and act as an anti-inflammatory agent. During DHCA procedures, ulinastatin can decrease the cytokine levels and improve the prognosis of the patients^[4]^. As a highly selective α_2_-adrenergic agonist, dexmedetomidine has analgesic, sedative, antianxiety, stress-reducing, and sympathetic nerve activity-inhibiting effects. It shows mild inhibition to respiration. In recent years, it has been widely used in patients during the perioperative period. Dexmedetomidine could inhibit the synthesis of the related inflammatory cytokines, such as TNF-α, IL-1, and IL-6 by inhibiting the Toll-like receptor 4/nuclear factor-κB signaling^[4]^. The infusion of antithrombin may prevent from further damage to the endothelial cells. In this way, the release of the inflammatory cytokines and the adhesion molecules were well controlled. It also demonstrated that the antithrombin possesses anti-inflammatory and anti-cellular adhesion effects^[10]^.

Pulmonary static inflation with 50% xenon during cardiopulmonary bypass decreased the oxygen index and increased the respiratory index values at the end of surgery for Stanford type A AD^[9]^.

The *Qishen Yiqi* dripping pill is made from the extracts with the active ingredients of *Radix Astragali, Radix Salviae Miltiorrhizae*, and *Radix Notoginseng* by modern pharmaceutical technologies^[28]^. *Qishen Yiqi* dripping pills have anti-inflammatory, antifibrotic, free radical scavenging, lipid-lowering, atherosclerotic plaque stabilizing, and tissue damage repairing effects. The inhibition of the inflammatory factors and the reduction of the inflammatory responses were also confirmed by clinical studies. Chen et al.^[29]^ applied the *Qishen Yiqi* dripping pills in patients with acute myocardial infarction for 12 weeks and found out that the serum brain natriuretic peptide, TNF-α, and IL-6 levels in the patients of the investigational treatment group significantly decreased in comparison to those of patients of the conventional treatment groups. It demonstrated that the *Qishen Yiqi* dripping pills could reduce serum TNF-α and IL-6 levels. The mechanism of action was considered to be related to the inhibition of inflammatory factors by the *Qishen Yiqi* dripping pills^[21]^. The Chinese medicine *Qishen Yiqi* dripping pills could lower serum inflammatory factors through the promotion of vascular endothelial cell repair, clearing the free radicals so as to enhance aortic vascular regeneration^[21]^.

Neutropenia is a common complication of chemotherapy in cancer. Pegfilgrastim, a granulocyte colony stimulating factor that stimulates bone marrow and promotes growth of the neutrophils, is effective in reducing the incidence of infection during chemotherapy associated with fever and neutropenia. Drug-induced aortitis caused by pegfilgrastim observed by Sato et al.^[17]^ was associated with an extremely elevated IL-6 on day 13 (714 pg/mL), while aortitis was found on day 14, and Stanford type B AD was incidentally found on day 36. The authors suggested a casual relation between pegfilgrastim, IL-6, and AD.

It has been proved in a series of experimental studies the crucial roles that IL-6 plays in the development of AD. Ju et al.^[30]^ revealed that AD was triggered by IL-6 signaling pathway and activator of transcription-3 via the Th17 lymphocyte-IL-17 axis. Sano and Anzai^[31]^ proved that the chemokine-dependent signaling caused neutrophilia and massive neutrophil accumulation in the dissected aorta, thereby leading to aortic enlargement and rupture via IL-6 production. Tieu et al.^[32]^ noted that inflammatory cytokines, including IL-6, were seen mainly in the tunica adventitia with local monocyte recruitment and activation, thereby promoting monocyte chemoattractant protein-1 (MCP-1) secretion, vascular inflammation, extracellular matrix degradation, and aortic destabilization. Moreover, activation of IL-6-signal transducer and activator of transcription 3 signaling was found to be responsible for the aneurysmal dilation in mgR/mgR mice through aggravating extracellular matrix degradation due to an upregulated matrix metalloproteinase-9^[33]^. It has been therefore proposed IL-6 neutralization as novel therapeutic strategies to prevent development of aneurysmal formation and rupture in patients with acute AD^[31]^.

## CONCLUSION

AD is an inflammatory process with an elevated IL-6 level in comparison to hypertensive or healthy controls. Adverse events, such as ALI, PIS, and death, are associated with an elevated IL-6 level. Therapeutic interventions, such as antithrombin, dexmedetomidine, ulinastatin, 50% xenon, and the *Qishen Yiq*i dripping pill, aiming at attenuating the inflammatory status can significantly decrease the IL-6 level. Endovascular aortic repair can effectively control the inflammatory cytokines. SACP with DHCA during aortic arch replacement shows better neuroprotective effect with an improved IL-6 level of the cerebrospinal fluid. The present study proved that circulating IL-6 could be a reliable biomarker for the diagnosis of AD and for the evaluation of the therapeutic outcomes and the prognosis of AD patients. Therapeutic interventions aiming at attenuating the inflammatory status by IL-6 neutralization could effectively decrease the IL-6 level and thus reverse the progression of the disorder of AD patient.

**Table:** 

Author's roles & responsibilities	
SMY	Conception or design of the work; or the acquisition, analysis, or interpretation of data for the work; drafting the work or revising it critically for important intellectual content; final approval of the version to be published
